# Prefrontal cortex activation during a cognitive reappraisal task is associated with real-life negative affect reactivity

**DOI:** 10.1371/journal.pone.0202888

**Published:** 2018-08-24

**Authors:** Jojanneke A. Bastiaansen, Elise C. Bennik, Jan Bernard C. Marsman, Johan Ormel, André Aleman, Albertine J. Oldehinkel

**Affiliations:** 1 University of Groningen, University Medical Center Groningen, Department of Psychiatry, Interdisciplinary Center Psychopathology and Emotion Regulation, Groningen, the Netherlands; 2 Friesland Mental Health Care Services, Department of Education and Research, Leeuwarden, the Netherlands; 3 University of Groningen, Department of Psychology, Groningen, the Netherlands; 4 University of Groningen, University Medical Center Groningen, Department of Neuroscience, Neuroimaging Center, Groningen, the Netherlands; Southwest University, CHINA

## Abstract

The neural substrate of cognitive reappraisal has been well-mapped. Individuals who successfully downregulate negative affect (NA) by reshaping their thoughts about a potentially emotional situation show augmented activity in the prefrontal cortex (PFC), with attenuated activity in the amygdala. We performed functional neuroimaging with experience sampling to determine whether individual differences in brain activation correspond to differences in real-life NA. While being scanned, 69 female students (aged 18–25 years) were asked to perform a cognitive reappraisal task. In addition, repeated assessments (5/day, 14 days) of affect and minor events in real-life were conducted. Individual t-maps were created for an instructed downregulation contrast (downregulate negative–attend negative) and an uninstructed regulation contrast (attend negative–attend neutral). Mean beta values were extracted from a priori defined regions of interest in the bilateral amygdala and PFC and were correlated with three daily life NA measures: baseline (mean) NA, NA variability, and NA reactivity to negative events. Only one out of twelve correlations for the amygdalae was nominally significant, which did not survive correction for multiple comparisons. PFC activation in the instructed and uninstructed regulation contrasts explained approximately 10% of the variance in NA reactivity; stronger recruitment during the attend-negative condition was correlated with lower reactivity levels. The degree to which individuals spontaneously engage frontal clusters may be a critical aspect of real-life emotional reactivity. The findings of this study provide a partial external validation of the cognitive reappraisal task, suggesting that frontal brain activation during implicit task conditions may have the strongest connection with real-life behaviors.

## Introduction

“I suspect that when you have people do some artificial task and look at their brains, the strongest activity you’ll see is in the parts of the brain that are responsible for doing artificial tasks” Steven Pinker (extract from an interview transcript in the *Journal of Cognitive Neuroscience*, 1994)

The capacity to regulate emotions is a necessary ability, enabling individuals to respond appropriately to stressful experiences and to navigate their social worlds. The process model of emotion regulation suggests that regulative strategies can affect different stages of the emotion-generative process with varying consequences [1–2]. Cognitive reappraisal is a commonly used (and widely investigated) strategy for downregulating negative emotions and is deployed relatively early in the emotion-generative process before emotional responses are fully developed. By changing an individual’s thinking about a situation, cognitive reappraisal can decrease its emotional impact at a relatively early stage. This strategy is considered to be more effective in decreasing an emotional experience than those applied following the activation of emotional response tendencies (e.g., through the suppression of emotion-expressive behavior) [2–3].

In the past decade, the neural underpinnings of emotion downregulation have been well-mapped. Meta-analyses have shown that instructed downregulation of negative affect (NA) consistently increases activation in regions of the prefrontal cortex (PFC) supporting domain-general cognitive control processes and decreases activation in emotion-generative brain regions such as the amygdala [4–7]. An early functional neuroimaging (fMRI) study found that compared with suppression, cognitive reappraisal results in relatively early PFC responses [8]. However, more recent studies using event-related potentials have refuted this finding [9–10]. Studies do suggest that cognitive reappraisal has a stronger effect than suppression in reducing negative emotions (and amygdala activation), at a lower cost [3, 8–10]. Although there is consensus among researchers that cognitive reappraisal recruits cognitive control regions to modulate emotional responses in the amygdala, the question of whether this is accomplished through the ventromedial prefrontal cortex (vmPFC) or through modulation of semantic representations in the lateral temporal cortex continues to be debated [6].

Although cognitive reappraisal is most often studied as an explicit regulation strategy, it can be unintentional and automatically triggered (i.e., implicit emotion regulation [11]). Unconscious reappraisal is relatively effortless and has been found to effectively reduce emotional reactivity [12–13]. The prefrontal regions that support intentional downregulation of emotion may also be engaged during uninstructed modulation of emotions (e.g., [14]). For instance, Silvers and colleagues [15] found that the degree to which individuals recruited prefrontal regions when responding “naturally” to negative stimuli was related inversely to their trial-by-trial self-reporting of NA. This could reflect unconscious or spontaneous use of regulative strategies. Moreover, greater habitual use of reappraisal strategies has been linked to decreased amygdala activity and to increased prefrontal activity during uninstructed as well as instructed regulation conditions [16–17].

Neuroimaging studies are performed in a very unusual setting (i.e., with the participant’s head enclosed in an MRI scanner coil) with mostly artificial stimuli and tasks to carefully control the environment. To isolate processes that are related to the cognitive control of emotion, many neuroimaging studies include emotion regulation tasks in which participants are instructed to respond naturally to pictures, without explicitly attempting to alter their feelings (uninstructed regulation) or to downregulate their NA through reinterpretation of negative pictures (instructed downregulation) ([18], for a list of reappraisal studies see [6]). However, cognitive reappraisal is rarely triggered explicitly in daily life (perhaps with the exception of psychotherapy). Moreover, daily emotional triggers are usually much more complex than static experimental images. To better understand what a cognitive reappraisal task actually measures requires a consideration of its external validity. Thus, the question to be addressed is: Do individual differences in brain activation, triggered by an experimental task, represent individual differences in real-life emotional experiences?

The external validity of neuroimaging tasks can be investigated using brain markers to predict real-world outcomes [19]. Urry and colleagues [20] obtained preliminary evidence (n = 16) for an association between changes in PFC and amygdala activation during NA downregulation and diurnal patterns of salivary cortisol secretion determined within participants’ home environments. Larger changes in PFC and amygdala activation predicted more normative patterns, which could reflect better adaptive functioning of the hypothalamic-pituitary-adrenal (HPA) axis and, hence, more functional stress responses. To the best of our knowledge, no studies have addressed the external validity of cognitive reappraisal tasks using measures of emotional processes in daily life.

Application of the experience sampling method (ESM) enables repeated sampling of behaviors and experiences in real time within participants’ natural environments [21–22]. ESM can thus illuminate important characteristics of NA dynamics in daily life, such as baseline NA, NA variability, and NA reactivity. Baseline NA refers to the typical affective state of an individual, or the setpoint to which affect returns after an increase or decrease in reactivity to internal and external events [23]. NA variability refers to the moment-to-moment fluctuations of affect, and NA reactivity represents NA fluctuations reflecting reactions to minor negative events (i.e., daily stressors).

In this study, we combined fMRI and ESM to examine whether activation in the PFC and the amygdala during a cognitive reappraisal task is associated with NA dynamics in daily life. We hypothesized that individuals demonstrating stronger amygdala activation in response to negative emotional stimuli and reduced amygdala deactivation during instructed downregulation show higher baseline NA, more NA variability, and higher NA reactivity in daily life. In addition, we hypothesized that stronger activation of frontal regulation clusters is related to generally lower NA levels, more stable NA, and smaller effects of negative events on NA. We did not posit differential hypotheses for the different areas within the distributed cognitive control network [4].

To decrease the number of potentially confounding factors, we restricted our study to female participants, thereby increasing the power of the study. Sex differences have been found not only in relation to the deployment of emotion regulation strategies (for a review see [24]) but also in the neural correlates of emotion processing and emotion regulation ([25]). These differences might put women at a higher risk for developing affective disorders [26].

## Materials and methods

### Study design

Data used in this study were derived from the Uncovering the Positive Potential of Emotional Reactivity (UPPER) study. This study comprised two parts: (1) an ESM study in which participants responded to questions on mood and context five times a day during fourteen consecutive days, and (2) an fMRI study in which two emotional tasks were administered and anatomical and resting state scans were conducted. This article reports on the cognitive reappraisal task, which was the first task performed during the fMRI session. The UPPER study was approved by the Medical Ethical Committee of the University Medical Center Groningen.

### Participants

Participants in the study were female students aged 18–25 years in Groningen (the Netherlands), recruited from the University of Groningen and Hanze University of Applied Sciences. Seventy-five right-handed female students participated in the ESM component of the study. Of these students, 71 (95%) completed more than 60 measurements, fixed as the a priori defined cut-off point and were enrolled in the fMRI study. The results for two participants were excluded from the analysis because of a technical error that occurred during MRI data acquisition (n = 1) and excessive motion (volume censoring exceeded 5%) during the task (n = 1). Thus, the final sample comprised 69 participants with a mean age of 20.79 years (SD = 1.84). Given that our study focused exclusively on women, it is important to note that most participants used oral contraceptives (n = 57). A minority used another hormone-releasing contraceptive (n = 4) or no contraceptive (n = 8). Of the non-contraceptive users, six were scanned during the follicular phase of their menstrual cycles.

To ensure a representative spread in daily life NA measures, participants were selected from a large sample of 268 students based on their scores for the 12-item neuroticism scale of the NEO Five-Factor Inventory [27]. Our selection procedure [28] resulted in a normal distribution of neuroticism scores (mean = 133.84, SD = 21.33) after reassessment using the 48-item neuroticism scale of the Revised NEO Personality Inventory [27]. None of the participants reported any past or current psychiatric disorders, or MRI contraindications (e.g., metal implants or claustrophobia), or used medication that could influence task effects. All participants were native Dutch speakers, had normal hearing, normal or corrected-to-normal vision, and provided written informed consent to participate in the study. Participants received financial compensation for their participation in the ESM and fMRI studies.

### Measures of negative affect in daily life

Details of the methods applied in the ESM study have been published previously [28]. ESM measurements were obtained through personal digital assistants using the PsyMate technology developed at Maastricht University [22] or through smartphones via a web-based software application for routine outcome monitoring (ROQUA, www.roqua.nl). ESM measurements, conducted during a 14-day period, were scheduled at 3-hour intervals at fixed time points during participants’ waking hours. At each time point, participants were asked to indicate which of the ten specified stressors they had experienced in the preceding 3-hour period. Accordingly, a dichotomous negative event (NE) variable was created, which indicated whether a stressor had occurred (0 = none, 1 = at least one). Each participant’s momentary negative affect (NA) was measured at each time point by averaging six NA items (“upset,” “irritated,” “nervous,” “listless,” “down,” and “bored”) that were rated using a 7-point scale ranging from 1 (“not at all”) to 7 (“very”). Missing values were assigned through multiple imputations entailing 15 iterations. Internal consistency for the NA scale (calculated across all time points and participants) was high (Cronbach’s alpha = 0.79). Three summary measures for each participant were derived from their NA scores. First, baseline NA was calculated by averaging NA across all time points. Second, NA variability was determined by calculating the root mean square of the successive differences (RMSSD) of NA. Third, NA reactivity was operationalized as the unstandardized regression coefficient derived from individual regression analyses, with NA as the dependent variable and the presence of a NE as an independent variable. To measure changes in NA resulting from the presence of a NE, the previous NA measurement (t-1) was included as an additional independent variable. The NA reactivity measure for one individual could not be determined because of the absence of reported negative events (NE occurred in only 3.2% of measurements). Thus, analyses of the NA reactivity measure were conducted for 68 (not 69) participants.

### Cognitive reappraisal task

The cognitive reappraisal task (adapted from [18, 29] entailed five different task conditions: attending to (1) neutral, (2) negative, or (3) positive images, (4) reappraising a negative image to downregulate NA, or (5) reappraising a positive image to upregulate PA. Here, we focus on the neutral and negative task conditions. Prior to being scanned, participants were trained on how to regulate their emotional responses and operate the panel buttons to self-report affect measures. For the downregulated negative condition, participants were instructed to decrease their emotional responses by viewing the situation as unreal or imagining an outcome for the scenario that differed from the suggested one.

Participants completed 110 trials, in series of 10, separated by 20-second fixation blocks. Equal numbers of trials for each condition were shown. Trials were presented in an event-related manner and lasted 15.5 seconds. The image stimulus was presented for 8 seconds in total. Two seconds after the stimulus appeared, a symbol appeared in the middle of the screen (1 s), instructing participants to stay attentive or to regulate their emotions actively over the next 5 seconds. After viewing each picture, participants had 3 seconds to rate the intensity of their emotions on a 7-point scale ranging from -3 (very negative) to +3 (very positive), followed by 4 seconds of rest (a “relax” message) and a black screen signaling the start of the next trial (0.5 s). The task was programmed using E-Prime (Psychology Software Tools, Pittsburgh, PA). The stimulus set comprised 22 neutral images (valence: M = 5.04, SD = 1.07; arousal: M = 2.70, SD = 1.81), 44 positive images (valence: M = 7.98, SD = 1.35; arousal: M = 5.42, SD = 2.45), and 44 negative images (valence: M = 2.05, SD = 1.33; arousal: M = 5.63, SD = 2.21) obtained from the International Affective Picture System [30]). Each stimulus was presented only once.

### fMRI data acquisition

Brain imaging data were obtained using a 3.0 Tesla MRI scanner (Philips Medical Systems, Best, the Netherlands), equipped with a 32-channel SENSE head coil. Functional images were obtained using a T2*-weighted echo-planar sequence with 37 axial slices recorded in descending order (voxel size = 3.5 × 3.5 × 3.5 mm, repetition time = 2000 ms, echo time = 20 ms, field of view = 224 × 129.5 × 224 mm, 64 × 62 in-plane matrix, flip angle = 70 degrees). Images were tilted 30° from the transverse plane of the anterior and posterior commissures to reduce artifacts from the nasal cavity. In addition, a shimbox was placed on the orbitofrontal regions. High-resolution T1-weighted structural images were obtained containing 170 slices (voxel size = 1 × 1 × 1 mm, repetition time (TR) = 9 ms, echo time (TE) = 8 ms, field of view = 232 × 170 × 256 mm, 256 × 256 in-plane matrix).

### Preprocessing and first and second-level analyses of fMRI data

All image processing was performed using the Statistical Parametric Mapping (Version 8) software package (SPM8; Wellcome Department of Cognitive Neurology, London, UK; http://www.fil.ion.ucl.ac.uk) in MATLAB R2009a (Version 7.8; The MathWorks, Inc., Natick, MA). Data preprocessing comprised the following steps: realignment to correct for subject motion, coregistration of the functional images on to the T1 anatomical image, spatial normalization into a standard space using a T1 template (Montreal Neurological Institute [MNI]), and smoothing with an isotropic Gaussian kernel (8-mm full width at half maximum) to minimize noise and accommodate residual neuroanatomical variations between participants.

Task regressors were analyzed at the subject level using boxcar functions convolved with the hemodynamic response function after applying 128-s high-pass filtering to remove low-frequency noise and slow drifts in the signal. For the first-level models, separate regressors were developed for the presentation of stimuli (8 s) for each of the five trial types. In addition, the rating and relax portions of each trial were modeled as two separate regressors. Head movements were accommodated through six motion regressors and their first temporal derivatives. To account for variability in the quality of single‐subject whole‐brain functional volumes, we used the Artifact Detection Toolbox (www.nitrc.org/projects/artifact_detect) to censor volumes with motion or intensity artifacts [31]. Volumes were censored when scan-to-scan movements exceeded 2 mm translation or 2° rotation in any direction and/or when the mean signal intensity per volume departed more than 4 standard deviations from the mean signal of all volumes in the time series [32]. Participants with censored volumes exceeding 5% were excluded from further analysis.

A voxel-by-voxel t-map of the instructed downregulation contrast (downregulate negative–attend negative) and the uninstructed regulation contrast (attend negative–attend neutral) was computed for each participant. Next, one-sample t-tests were performed at the second level to determine task effects at the group level. The t-maps and con images of these second-level whole-brain analyses are provided in the Supporting Information (S1 Dataset) to benefit future meta-analyses. Our focus in this study was on correlations between brain activation in a priori defined regions of interest (ROIs) and daily life NA measures.

### Brain measures

**Fig 1:**
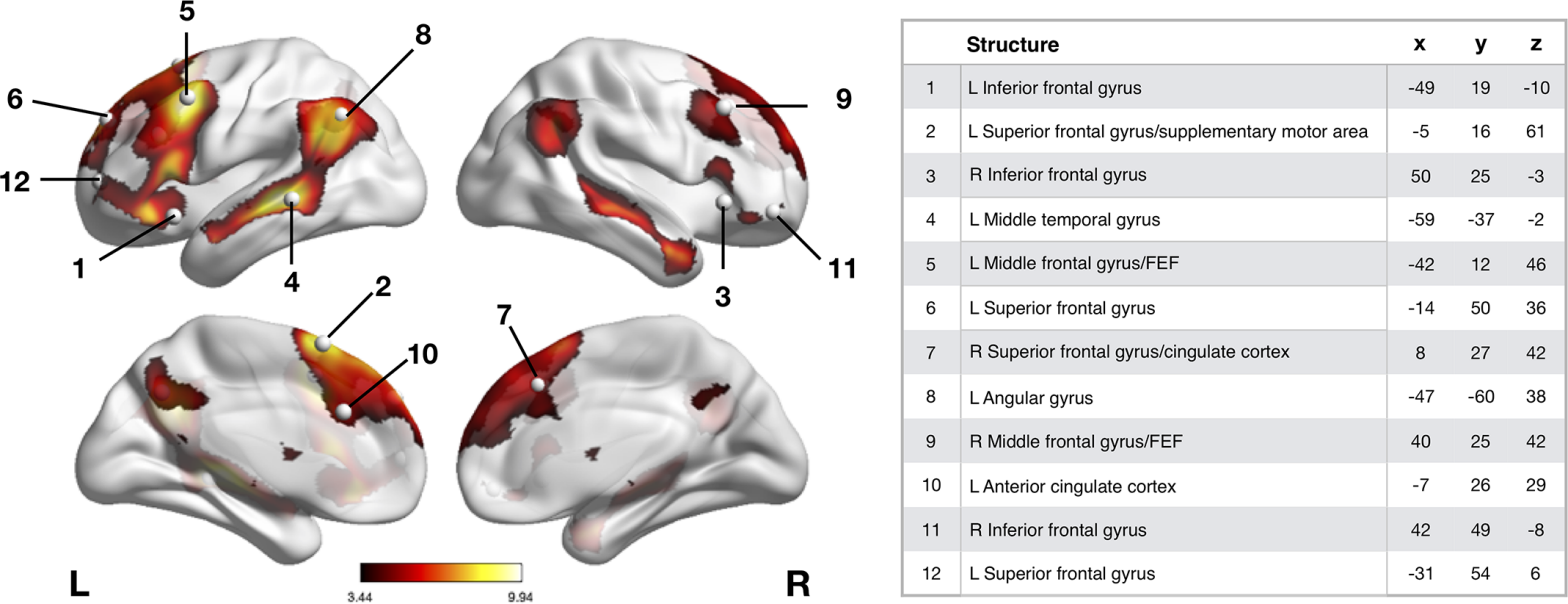
Downregulation task map and regions of interest. The threshold (T) value for the SPM T-map of the instructed downregulation contrast was 3.12 (uncorrected p < .001). The gray dots in Fig 1 represent peak coordinates of the regulation clusters derived from [4], which are listed with their corresponding MNI coordinates. Results were visualized using BrainNet Viewer [36].

Twelve spherical 5 mm PFC ROIs (Fig 1) were defined based on the peak coordinates of clusters that consistently featured in NA downregulation, as identified in a recent quantitative meta-analysis covering 963 participants across 44 studies on emotion downregulation (Table 3 in [4]). For each ROI, we calculated two different subject-specific measures: (1) downregulation, defined as the average decrease in activation during instructed NA downregulation compared with the attend-negative condition (instructed downregulation contrast) and (2) reactivity, defined as the participant’s average response to negative stimuli compared with neutral stimuli (uninstructed regulation contrast). Subsequently, overall downregulation and reactivity measures for PFC were created by averaging values across the 12 ROIs.

We defined separate ROIs for the right and left amygdala, applying the AAL template of the WFU PickAtlas (Version 3.0 [33,34]). This is because peak coordinates for amygdala downregulation vary widely across meta-analyses and hence, reappraisal-related activation could be easily overlooked. To prevent distortions of the correlations between brain activation and daily life NA measures caused by inactive voxels, we applied subject-specific masks, which retained only active voxels within the ROIs of each participant. For both ROIs, we developed individual downregulation and reactivity measures based on the mean beta values for the respective instructed downregulation and uninstructed regulation contrasts.

### ESM-fMRI analysis

The brain measures and daily life NA measures for each individual are presented in the S2 Dataset. We performed separate Pearson correlation analyses between the two brain measures (reactivity and downregulation) and the three daily life NA measures (baseline NA, NA variability, and NA reactivity) for the left and right amygdalae. A p-value below .05 was considered statistically significant for all 12 analyses. For the PFC regulation clusters, we performed six correlational analyses (3 daily life NA measures × 2 brain measures) for the overall measures, applying an α value of .05. Because we were performing multiple statistical tests for the amygdalae and PFC, we interpreted the general pattern of associations as opposed to each individual effect (which could lead to capitalization on chance). That is, we considered the proportion of significant associations compared with the total number of tests conducted for the amygdalae and the PFC regulation clusters, respectively. We formalized this approach by applying the false discovery rate (FDR) method [35] to correct for multiple comparisons (the maximum acceptable FDR value was set at .05). Analyses were performed with SPSS 23 (SPSS Inc., Chicago, IL).

## Results

### Descriptives

#### Affect ratings

In the ESM study, baseline NA values ranged from 1.1 to 4.2 (on a 1–7 scale), with an average value of 1.76 (SD = 0.58). The mean NA variability (RMSSD) was 0.72 (SD = 0.24). Mean NA reactivity was 0.35, with a standard deviation of 0.23. Thus, on average, the occurrence of a negative event predicted an increase of 0.35 points for NA compared with the NA level at the preceding time point. The standard deviation indicates that the degree to which participants were affected by the occurrence of negative events varied. During the cognitive reappraisal task performed in the MRI scanner, NA values could range from -3 to 0 (on a 7-point scale extending up to +3). Affect ratings for attend-negative trials (M = -1.46, SD = 0.51) were significantly lower than those for attend-neutral trials (M = 0.17, SD = 0.27, t(68) = -24.49, p < .001). Affect ratings for downregulated negative trials (M = -1.17, SD = 0.62) were significantly higher than those for attend-negative trials (t(68) = 3.84, p < .001).

#### fMRI measures

Neither amygdala (left or right) was significantly affected by the instructed downregulation contrast (right: t(68) = .81, p = .43; left: t(68) = .46, p = .65). By contrast, the amygdala ROIs were significantly more affected by negative stimuli than by neutral stimuli (i.e., uninstructed regulation contrast, right: t(68) = 2.51, p < .05; left: t(68) = 2.71, p < .01).

Fig 1 shows that our a priori defined PFC downregulation clusters, derived from a meta-analysis conducted by Frank and colleagues [4], were contained within our instructed downregulation task map. These ROIs were evidently more strongly activated by downregulation than by the attend-negative condition. In fact, one-sample t-testing of participants’ average beta values for the downregulation contrast (all *p* < .001) showed that all 12 ROIs were significantly more involved during downregulation than when participants attended to negative stimuli.

### Brain measures and NA in daily life

Table 1 shows the correlations between the brain activation measures and the daily life NA measures. For the amygdalae, only one of the twelve correlational analyses was significant at the nominal threshold: for the instructed downregulation contrast, activation of the left amygdala was related to NA reactivity in daily life (r = -.26). Thus, individuals demonstrating relatively lower decreases in amygdala activation when instructed to downregulate their NA (compared with the attend-negative condition) were more reactive to negative events in daily life. However, this association did not survive FDR-correction.

**Table 1:** Correlations between brain activation and daily life measures.

	NA baseline		NA variability		NA reactivity	
	R	P-value	R	P-value	R	P-value
**Right amygdala**						
Downregulation (r)	**-.09**	.45	**-.16**	.19	**-.11**	.37
Reactivity	**-.11**	.35	**-.05**	.70	**-.04**	.73
**Left amygdala**						
Downregulation (r)	**.01**	.93	**-.15**	.22	**-.26**	.03*
Reactivity	**.01**	.94	**-.03**	.80	**-.17**	.18
**Regulation clusters**						
Downregulation	**-.16**	.19	**.16**	.19	**.33**	.01*†
Reactivity	**-.04**	.75	**-.16**	.20	**-.31**	.01*†

Notes

* uncorrected p < .05

† p-value < a multiple test correction significance threshold of .017, r = reversed sign. To facilitate interpretation, a greater decrease in activation in the amygdala is represented by a more positive value for the instructed downregulation contrast.

For the PFC regulation clusters, the two correlational analyses of NA reactivity were significant at the nominal threshold and survived the FDR-derived significance threshold (p < .017). Approximately 10% of the variance in NA reactivity was explained by individual differences in the degree of recruitment of regulation clusters. Overall activation of the PFC regulation clusters for the instructed downregulation contrast was correlated *positively* with NA reactivity. Post-hoc analyses of the raw measures revealed the positive sign for this correlation resulted from the comparative condition (attend negative: r = -.28, versus downregulate negative: r = -.05). Overall activation of the regulation clusters for the uninstructed regulation contrast was correlated *negatively* with NA reactivity. Thus, the degree to which regulation clusters were recruited spontaneously when participants were confronted with negative images (compared to neutral images) was related to NA reactivity in daily life. Post-hoc analyses of the raw measures indicated that NA reactivity was related to PFC activation during both the attend-negative and attend-neutral conditions (r = -.30). S1 Fig depicts scatterplots of the correlations between NA reactivity and PFC activation during the three task conditions.

### Post-hoc analyses

#### Multilevel model for NA reactivity

To verify that significant associations were not methodological artifacts of correlational analyses, we reanalyzed the NA reactivity data. Accordingly, we applied a multilevel approach, accommodating the nested structure of the data (e.g. [37]), with time points (level 1) nested within individuals (level 2). We included NA as the dependent variable and the previous NA measurement (t-1) and NE as independent variables at level 1 (person-mean centered). Brain measures and their interactions with NE were included as person-based variables at level 2 (grand-mean centered). Details of the analysis are presented in S1 Appendix, and full models of the fixed effects are presented in S1–S3 Tables. The results were very similar to the correlational analyses. Downregulation of the right amygdala (*b* = −.11, *p* = .53) and its reactivity (*b* = −.04, *p* = .78) and reactivity of the left amygdala (*b* = −.23, *p* = .24) did not moderate the relationship between a NE and changes in NA (NA reactivity). The negative relationship between downregulation of the left amygdala and NA reactivity was no longer statistically significant (*b* = −.35, *p* = .07). However, the positive relationship between NA reactivity and downregulation of the regulation clusters (*b* = .58, *p* = .01) and the negative relationship between NA reactivity and reactivity of the regulation clusters (*b* = -.46, *p* = .04) remained significant.

#### Whole-brain correlation analyses

**Fig 2:**
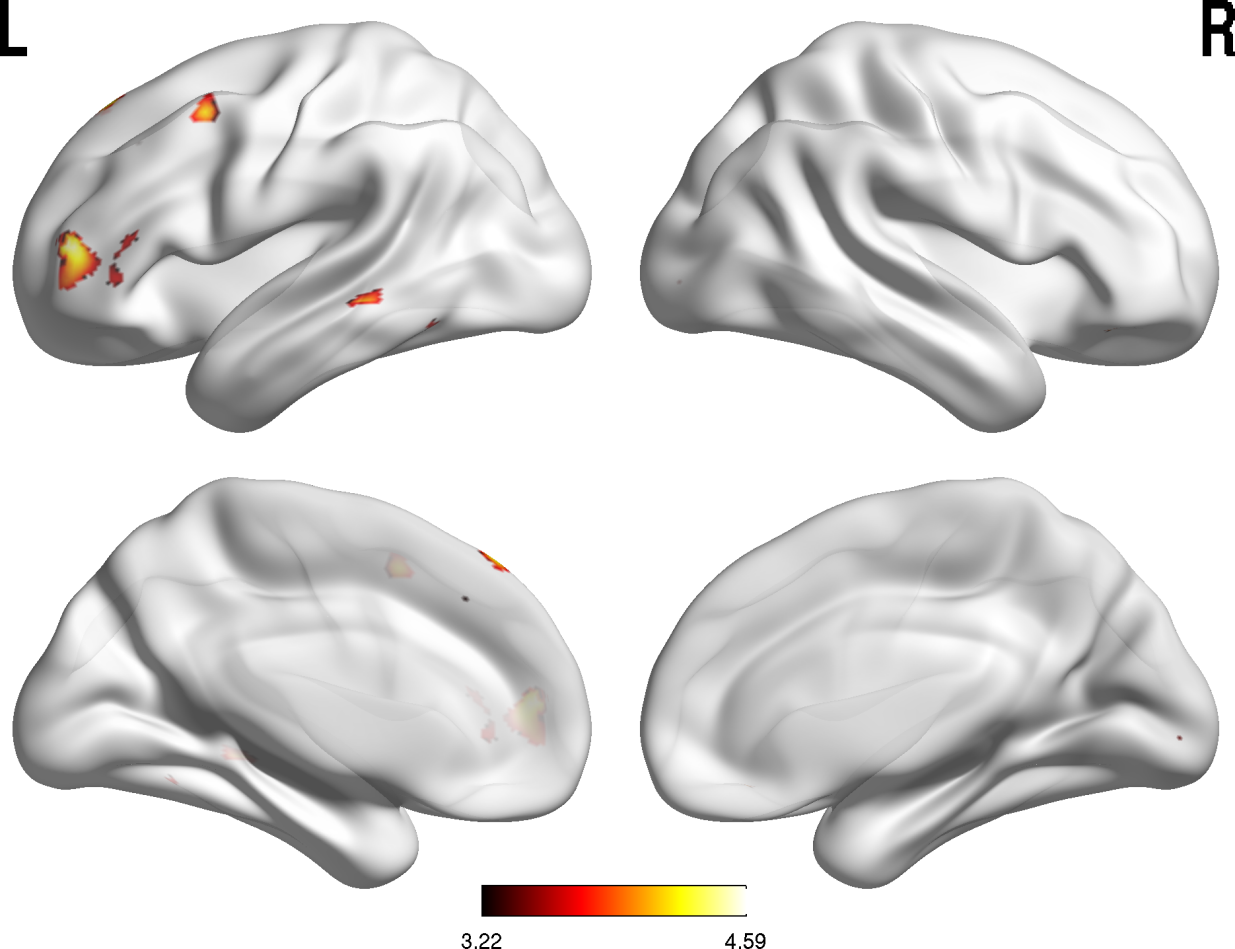
Whole-brain correlation analysis for NA reactivity during uninstructed downregulation. BrainNet Viewer [36] was used to visualize a SPM T-map of the correlation between NA reactivity and brain activation based on the instructed downregulation contrast and a T value of 3.22 (uncorrected p < .001).

Our work on individual differences in brain activation and real-life NA can inform hypothesis formulation and ROI selection in future studies. Therefore, we have included t-maps and con images of second-level whole-brain correlation analyses in the Supporting Information (S3–S5 Datasets). Following the suggestion of a reviewer of our original manuscript, we conducted a whole-brain analysis to assess the (negative) correlation between NA reactivity and brain activation during uninstructed regulation, which confirmed the involvement of our ROIs (Fig 2). Notably, NA reactivity in daily life was correlated negatively with brain activation in clusters in the left inferior frontal gyrus, left middle frontal gyrus, and left middle temporal gyrus.

#### Habitual use of cognitive reappraisal

Our main analyses showed that the degree to which the frontal regulation clusters are spontaneously (or unconsciously) recruited by individuals when they are confronted with negative images is related to individual differences in daily life NA reactivity. Following the suggestion of a reviewer, we examined how these findings were related to habitual cognitive reappraisal strategies deployed in daily life. We found that habitual cognitive reappraisal (as measured with the Emotion Regulation Questionnaire [38]) was correlated negatively with NA reactivity in daily life (r = -.28, p < .05) but not with the other daily life NA measures (baseline NA: r = -.08, p = .52; NA variability: r = -.06, p = .60). Thus, NA reactivity in daily life appears to be related to habitual reappraisal. Moreover, we examined whether the pattern of results for individual differences in habitual reappraisal was the same as that for individual differences in NA reactivity. That is, we investigated whether habitual reappraisal was correlated more strongly with the degree to which regulation clusters were spontaneously recruited when participants were confronted with negative images compared with their deployment of regulation clusters when instructed to use reappraisal to downregulate their emotional responses. There was no correlation between habitual reappraisal and PFC activation either for the attend-negative condition (r = -.04, p = .72) or for the downregulated negative condition (r = .01, p = .97). Therefore, although NA reactivity was associated with habitual reappraisal in our study, its association with PFC activation was stronger.

## Discussion

We combined functional neuroimaging and ESM to examine the relationship between brain activation during a cognitive reappraisal task and emotional daily life processes. Our data did not support the hypothesized links between amygdala activation and NA dynamics in daily life. However, an association between the recruitment of frontal regulation clusters and NA reactivity in daily life was supported.

Specifically, our hypothesis that individuals whose amygdalae respond more strongly to negative emotional stimuli show stronger NA responses to negative events, or have generally higher NA intensity in daily life, was not supported. Moreover, the hypothesis that individuals who are less able to downregulate amygdala activation in response to negative stimuli have generally higher baseline levels of NA in daily life and less stable NA was also not supported by the results. However, we found a significant negative correlation between left amygdala downregulation and NA reactivity to negative events in daily life. This finding endorses that of a recent study, which reported a positive relationship between amygdala activation and trial-to-trial fluctuations in NA during a cognitive reappraisal task [15]. However, our result could be a random finding, given that the p-value for the daily life association in our study was only marginally significant at the nominal level, did not survive correction for multiple comparisons, and did not reach significance in the multilevel analysis. Moreover, a similar correlation was not found for the right amygdala. Thus, the relationship between NA reactivity and amygdala activation did not convincingly extend beyond the confines of the laboratory.

For the frontal regulation clusters, we hypothesized that stronger recruitment relates to generally lower NA levels, more stable NA, and lower NA reactivity in daily life. Our findings did not support linkages between PFC activation and baseline NA or NA variability. However, they did indicate a positive correlation between overall activation of the regulation clusters in the instructed regulation contrast and NA reactivity and a negative correlation between activation in the uninstructed regulation contrast and NA reactivity. The direction of these findings appears contradictory. Post-hoc tests revealed that the differential effects were driven by a negative correlation existing between NA reactivity and the attend-negative condition, which underlies both contrasts. In fact, the degree to which the frontal regulation clusters were spontaneously recruited by individuals when they were confronted with negative images explained 10% of the variance in daily life NA reactivity. The possibility that this is a random finding cannot be ruled out. However, the results survived correction for multiple comparisons and were robust across different modelling approaches. Moreover, of the real-life measures, NA reactivity to negative events seems to be related most closely to the neural responses evoked by emotional events in the MRI scanner. Furthermore, this finding supports the idea that regulation of emotions in daily life is less about the ability to regulate emotions under conditions of prompting and more about whether these skills are deployed spontaneously [11]. In our study, individuals who demonstrated lower NA reactivity levels in daily life were more prone to routinely use cognitive reappraisal. It is possible that these individuals intentionally applied reappraisal during our task, even in the absence of instructions to do so (i.e. in the attend-negative condition). Alternatively, these individuals may have unconsciously engaged in implicit regulation strategies [39]. A previous study by Drabant and colleagues [17] showed that higher levels of habitual reappraisal in everyday life were related to increased prefrontal and parietal activity (and decreased amygdala activity) during the processing of negative emotional facial expressions. Our findings indicated that although NA reactivity was associated with habitual cognitive reappraisal, it was more closely related to PFC activation.

Emotion regulation tasks are designed to isolate processes that relate to intentional cognitive control of emotions. These paradigms have been used to map abnormalities in emotion regulation neural circuitry in psychiatric disorders such as depression in the hopes of shedding light on their pathogenesis [40]. Our findings suggest that brain regions targeted by cognitive reappraisal tasks are involved in emotional daily life processes. Further, activation of these brain regions during uninstructed conditions may better capture real-life differences in emotional processing than activation during instructed regulation conditions. Thus, a cognitive reappraisal task could be used when attempting to identify regulation regions, but more implicit tasks may be appropriate for mapping emotion regulation difficulties in psychiatric disorders. Researchers have posited that spontaneous use of regulation strategies, as opposed to the ability to deploy these strategies with prompting, is integral to psychopathology, but this hypothesis requires more extensive testing (e.g., [41]).

This study was the first to relate brain activation during a cognitive reappraisal task to emotional daily life processes. Our pursuit of a hypothesis-driven approach and our selection of only regions demonstrated to be strongly implicated in emotion regulation in a recent meta-analysis [4] were strengths of the study. All implicated regions were activated during the performance of our task. A limitation of the study was that coverage of the vmPFC was not optimal. It has been suggested that the vmPFC plays an important role in emotion regulation, but only a minority of studies (e.g. [7]) have demonstrated its significant activation. This could be attributed to variations in experimental designs but also to signal loss in its basal parts, as evidenced in our study. Therefore, it remains unclear how vmPFC activation relates to NA dynamics in daily life. Another limitation of the study is that we focused exclusively on healthy young women (to restrict the number of potentially confounding variables). Hence, we do not know whether our results are generalizable to men, older individuals, and clinical populations. Women have been reported to be more susceptible to negative emotions than men (e.g. [42]), and studies have revealed sex differences in brain structure and function (e.g. [43–44]). However, recent research suggests that these differences may not be as pronounced as the literature suggests (e.g. [45]). Moreover, the majority of our sampled participants used oral contraceptives, which are known to have a mood-stabilizing effect [46].

In sum, the external validity of fMRI tasks is often considered to be low because of the artificial nature of the stimuli and task instructions, the need for repetition, and the constrained setting. We have shown that frontal brain activation during an artificial emotion regulation task does relate to real-life emotional reactivity (but not to baseline NA or NA variability). The degree to which frontal clusters are spontaneously engaged by individuals may be central to the relevance for everyday life. This study provides a partial external validation of cognitive reappraisal tasks and suggests that frontal brain activation during implicit task conditions may have the strongest connection with real-life behaviors. If replicated, these findings may have important implications for the interpretation of cognitive reappraisal tasks.

## Supporting information

S1 Dataset Second-level whole-brain analyses. T-maps and con images obtained for second-level whole-brain analyses (instructed downregulation contrast, i.e., downregulate negative–attend negative and uninstructed regulation contrast, i.e., attend negative–attend neutral).
(ZIP)

S2 Dataset Brain and daily life NA measures. Imputed dataset comprising brain measures and daily life NA measures for each individual.
(SAV)

S3 Dataset Second-level whole brain correlational analyses of baseline NA. T-maps and con images obtained for second-level whole-brain correlational analyses of baseline NA (instructed downregulation contrast, i.e., downregulate negative–attend negative and uninstructed regulation contrast, i.e., attend negative–attend neutral).
(ZIP)

S4 Dataset Second-level whole brain correlational analyses of NA variability. T-maps and con images obtained for second-level whole-brain correlational analyses of NA variability (instructed downregulation contrast, i.e., downregulate negative–attend negative and uninstructed regulation contrast, i.e., attend negative–attend neutral).
(ZIP)

S5 Dataset Second-level whole brain correlational analyses of NA reactivity. T-maps and con images obtained for second-level whole-brain correlational analyses of NA reactivity (instructed downregulation contrast, i.e., downregulate negative–attend negative and uninstructed regulation contrast, i.e., attend negative–attend neutral).
(ZIP)

S1 Fig Scatterplots of correlations between NA reactivity and PFC activation during the three task conditions. R^2^ = explained variance.
(DOCX)

S1 Appendix Details of the multilevel regression analysis. (DOCX)

S1 Table Multilevel regression results for the right amygdala. NA_t-1_ = negative affect at the previous measurement (t-1), NE = negative event (dichotomous variable), r = reversed sign. To facilitate interpretation, a greater decrease in activation in the amygdala is represented by a more positive value for the instructed downregulation contrast.
(DOCX)

S2 Table Multilevel regression results for the left amygdala. NA_t-1_ = negative affect at the previous measurement (t-1), NE = negative event (dichotomous variable), r = reversed sign. To facilitate interpretation, a greater decrease in activation in the amygdala is represented by a more positive value for the instructed downregulation contrast.
(DOCX)

S3 Table Multilevel regression results for the regulation clusters. NAt-1 = negative affect at the previous measurement (t-1), NE = negative event (dichotomous variable).
(DOCX)
