# Supplementary material for: Prefrontal cortex activation during a cognitive reappraisal task is associated with real-life negative affect reactivity
Source: PLoS One. 2018 Aug 24;13(8):e0202888. doi: 10.1371/journal.pone.0202888 (PMC6121771; doi:10.1371/journal.pone.0202888)
Supplement: S1 Fig — R2 = explained variance. (DOCX) [file pone.0202888.s006.docx]

**S1 Fig. Scatterplots of correlations between NA reactivity and PFC activation during the three task conditions.**


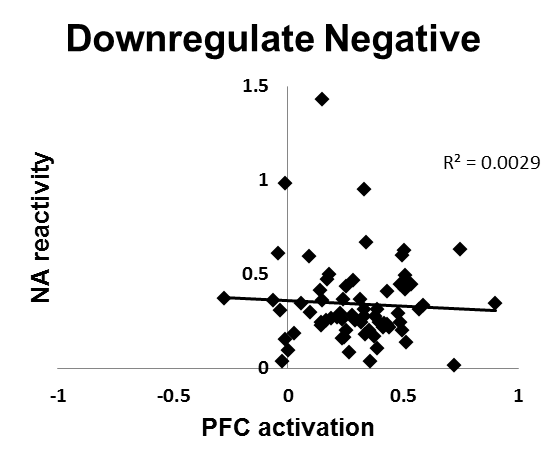

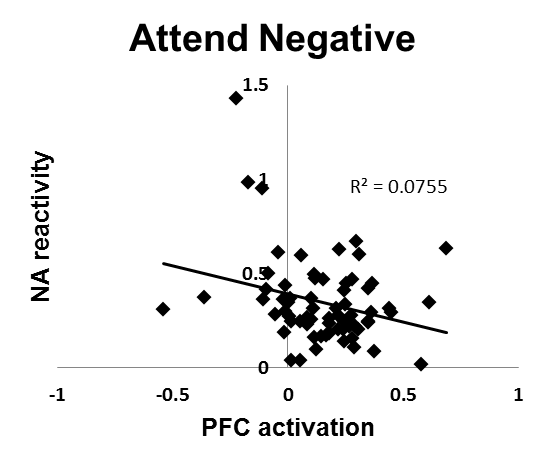

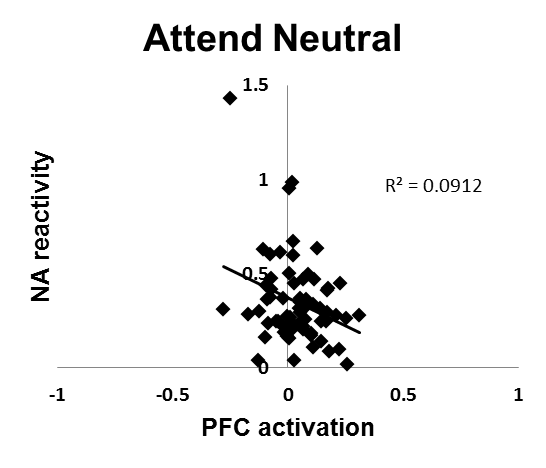


Note: R^2^ = explained variance.
