# Supplementary material for: Prefrontal cortex activation during a cognitive reappraisal task is associated with real-life negative affect reactivity
Source: PLoS One. 2018 Aug 24;13(8):e0202888. doi: 10.1371/journal.pone.0202888 (PMC6121771; doi:10.1371/journal.pone.0202888)
Supplement: S1 Appendix — (DOCX) [file pone.0202888.s007.docx]

**S1 Appendix. Details of the multilevel regression analysis.**

For negative affect (NA) reactivity we applied a multilevel approach, which accommodates the nested structure of the data (e.g. Lopez et al., 2014): time points (level 1) nested within individuals (level 2). We included NA as the dependent variable, and NA at the previous measurement (t-1) and the dichotomous negative event (NE) variable as independent variables at level 1. Brain measures and their interactions with NE were included as person-based variables at level 2 (grand-mean centered).

The mathematical model is outlined below:

Level 1:
NA_ij_ = β_0j_ + β_1j_NA_t-1_ + β_2j_NE + r_ij_

in which NA_ij_ is the NA score for person j on moment i, β_0j_ is a coefficient representing the intercept for person j, β_1j_ NA_t-1_ is a coefficient (a slope) for NA_t-1_, β_2j_ NE is a coefficient (a slope) for NE, and r_ij_ represents error.

Level 2:

β_0j_ = γ_00_ + γ_01_ (brain measure) + u_0j_;
β_1j_ = γ_10_ + γ_11_ (brain measure) + u_1j_;
β_2j_ = γ_20_ + γ_21_ (brain measure) + u_2j_.

in which γ_00_, γ_10_ and γ_20_ represent the average of the intercept, the NA slope and the NE slope, respectively. γ_01_, γ_11_, γ_12_ represent the moderating effect of the person-level brain measure variable (e.g. right amygdala downregulation).

All three within-person coefficients were modeled as random (the terms u_0j_, u_1j_, and u_2j_ were included), implying that the strength of the intercept and the two slopes were allowed to vary across individuals.

In line with the recommendation described in Wang and Maxwell (2015), we used person-mean centering for the level 1 variables NA_t-1_ and NE, and did not apply detrending before analyses, because there was no indication of a time effect. Level 2 variables were grand mean centered. We used an unstructured covariance structure to model variance. The analyses were performed in SPSS with restricted maximum likelihood estimation.

Literature:

Lopez RB, Hofmann W, Wagner DD, Kelley WM, Heatherton TF. Neural predictors of giving in to temptation in daily life. Psychol Sci*.* 2014;25: 1337-1344.

Wang L, Maxwell SE. On disaggregating between-person and within-person effects with

longitudinal data using multilevel models. Psychol Methods. 2015;20: 63–83.
