# Supplementary material for: Prefrontal cortex activation during a cognitive reappraisal task is associated with real-life negative affect reactivity
Source: PLoS One. 2018 Aug 24;13(8):e0202888. doi: 10.1371/journal.pone.0202888 (PMC6121771; doi:10.1371/journal.pone.0202888)
Supplement: S1 Table — NAt-1 = negative affect at the previous measurement (t-1), NE = negative event (dichotomous variable), r = reversed sign. To facilitate interpretation, a greater decrease in activation in the amygdala is represented by a more positive value for the instructed downregulation contrast. (DOCX) [file pone.0202888.s008.docx]

**S1 Table. Multilevel regression results for the right amygdala.**

| **Downregulation** | *b* | SE | *p* | **Reactivity** | *b* | SE | *p* |
| --- | --- | --- | --- | --- | --- | --- | --- |
| Level 1 predictors |  |  |  | Level 1 predictors |  |  |  |
| Intercept | 1.76 | .07 | .00 | Intercept | 1.76 | .07 | .00 |
| NA_t-1_ | .17 | .02 | .00 | NA_t-1_ | .17 | .02 | .00 |
| NE | .33 | .03 | .00 | NE | .33 | .03 | .00 |
| Level 2 predictors |  |  |  | Level 2 predictors |  |  |  |
| Downregulation (r) | -.33 | .41 | .42 | Reactivity | -.40 | .35 | .25 |
| Downregulation x NE (r) | -.11 | .18 | .53 | Reactivity x NE | -.04 | .16 | .78 |

Note: NA_t-1_ = negative affect at the previous measurement (t-1), NE = negative event (dichotomous variable), r = reversed sign. To facilitate interpretation, a greater decrease in activation in the amygdala is represented by a more positive value for the instructed downregulation contrast.
