# Supplementary material for: Prefrontal cortex activation during a cognitive reappraisal task is associated with real-life negative affect reactivity
Source: PLoS One. 2018 Aug 24;13(8):e0202888. doi: 10.1371/journal.pone.0202888 (PMC6121771; doi:10.1371/journal.pone.0202888)
Supplement: S3 Table — NAt-1 = negative affect at the previous measurement (t-1), NE = negative event (dichotomous variable). (DOCX) [file pone.0202888.s010.docx]

**S3 Table. Multilevel regression results for the regulation clusters.**

| **Downregulation** | *b* | SE | *p* | **Reactivity** | *b* | SE | *p* |
| --- | --- | --- | --- | --- | --- | --- | --- |
| Level 1 predictors |  |  |  | Level 1 predictors |  |  |  |
| Intercept | 1.76 | .07 | .00 | Intercept | 1.76 | .07 | .00 |
| NA_t-1_ | .17 | .02 | .00 | NA_t-1_ | .17 | .02 | .00 |
| NE | .33 | .03 | .00 | NE | .33 | .03 | .00 |
| Level 2 predictors |  |  |  | Level 2 predictors |  |  |  |
| Downregulation | -.87 | .46 | .06 | Reactivity | .28 | .51 | .59 |
| Downregulation x NE | .58 | .22 | .01 | Reactivity x NE | -.46 | .22 | .04 |

Note: NA_t-1_ = negative affect at the previous measurement (t-1), NE = negative event (dichotomous variable)
